# A multiplexed assay for quantifying immunomodulatory proteins supports correlative studies in immunotherapy clinical trials

**DOI:** 10.3389/fonc.2023.1168710

**Published:** 2023-05-02

**Authors:** Jeffrey R. Whiteaker, Lei Zhao, Regine M. Schoenherr, Dongqing Huang, Rachel A. Lundeen, Ulianna Voytovich, Jacob J. Kennedy, Richard G. Ivey, Chenwei Lin, Oscar D. Murillo, Travis D. Lorentzen, Simona Colantonio, Tessa W. Caceres, Rhonda R. Roberts, Joseph G. Knotts, Joshua J. Reading, Candice D. Perry, Christopher W. Richardson, Sandra S. Garcia-Buntley, William Bocik, Stephen M. Hewitt, Shrabanti Chowdhury, Jackie Vandermeer, Stephen D. Smith, Ajay K. Gopal, Nirasha Ramchurren, Steven P. Fling, Pei Wang, Amanda G. Paulovich

**Affiliations:** ^1^ Clinical Research Division, Fred Hutchinson Cancer Center, Seattle, WA, United States; ^2^ Cancer Research Technology Program, Antibody Characterization Lab, Frederick National Laboratory for Cancer Research, Frederick, MD, United States; ^3^ Experimental Pathology Laboratory, Laboratory of Pathology, Center for Cancer Research, National Cancer Institute, National Institute of Health, Bethesda, MD, United States; ^4^ Department of Genetics and Genomic Sciences, Icahn School of Medicine at Mount Sinai, New York, NY, United States; ^5^ Division of Medical Oncology, Department of Internal Medicine, University of Washington, Seattle, WA, United States; ^6^ Vaccine and Infectious Disease Division, Fred Hutchinson Cancer Center, Seattle, WA, United States

**Keywords:** correlative biomarkers, immunotherapy, immuno-oncology, immuno-MRM, proteomics, targeted proteomics

## Abstract

**Introduction:**

Immunotherapy is an effective treatment for a subset of cancer patients, and expanding the benefits of immunotherapy to all cancer patients will require predictive biomarkers of response and immune-related adverse events (irAEs). To support correlative studies in immunotherapy clinical trials, we are developing highly validated assays for quantifying immunomodulatory proteins in human biospecimens.

**Methods:**

Here, we developed a panel of novel monoclonal antibodies and incorporated them into a novel, multiplexed, immuno-multiple reaction monitoring mass spectrometry (MRM-MS)-based proteomic assay targeting 49 proteotypic peptides representing 43 immunomodulatory proteins.

**Results and discussion:**

The multiplex assay was validated in human tissue and plasma matrices, where the linearity of quantification was >3 orders of magnitude with median interday CVs of 8.7% (tissue) and 10.1% (plasma). Proof-of-principle demonstration of the assay was conducted in plasma samples collected in clinical trials from lymphoma patients receiving an immune checkpoint inhibitor. We provide the assays and novel monoclonal antibodies as a publicly available resource for the biomedical community.

## 1 Introduction

The emergence of immunotherapies, such as immune checkpoint inhibitors, is revolutionizing cancer care (1–5). However, substantial responses are typically seen only in subsets of patients (6), and immune-related adverse events (irAEs) occur in a significant number of those receiving treatment (7). Improving upon current immunotherapy treatment modalities in cancer relies on developing biomarkers to predict and monitor irAEs, identifying biomarkers to select patients likely to respond to single-agent *vs*. combination immunotherapy (8), and understanding the mechanisms of response and resistance to immunotherapy to design more efficacious and less toxic treatments.

The immune response to cancer is driven by hundreds of immunomodulatory proteins in the tumor microenvironment and the periphery, called the “cancer-immunity cycle” (9, 10). Immunoassay platforms (e.g., immunohistochemistry, flow cytometry, enzyme-linked immunoassays) are used to measure protein expression for some of these targets; however, these platforms are dependent on monospecific antibodies, which often are not available, resulting in analytical issues, especially with assay interferences (11). Thus, complementary technologies for robust multiplexed quantification of immunomodulatory proteins are needed to better evaluate the cancer-immunity cycle and productively impact translational research.

To address this need, we recently established a multiplexed, mass spectrometry-based assay (the “IO-1 assay”) for the quantification of 46 immunomodulatory proteins and demonstrated the performance of the IO-1 assay in tissue and plasma biospecimens (12). The IO-1 assay is based on coupling monoclonal antibody-based immunoaffinity enrichment of peptides with targeted, multiple reaction monitoring (MRM) mass spectrometry in a technique called immuno-MRM (13). Immuno-MRM assays enable the precise quantification of low-abundance proteins (14, 15), standardization across laboratories (16), high multiplexing capability (17), highly specific measurements, and implementation in clinical laboratories (18, 19).

In this report, we extend our previous work by developing a new panel of monoclonal antibodies and configuring them into a novel, multiplex “IO-2” immuno-MRM assay that significantly expands our capability by enabling the quantification of additional immunomodulatory proteins (i.e., not included in the IO-1 panel) in tissues. The IO-2 assay targets 49 proteotypic peptides representing 43 proteins, was validated according to fit-for-purpose guidelines (20) in plasma and tissue matrices, and can be used in combination (i.e., on the same biospecimen) with the previously developed IO-1 panel. We applied the IO-2 assay to a panel of biospecimens (102 tissue biopsies and 48 plasma samples) obtained from cancer patients to characterize the expected performance of the assays in real-world samples. Additionally, we present proof-of-principle for the combined use of the IO-1 and IO-2 assay panels to support correlative studies in clinical trials of lymphoma patients receiving an immune checkpoint inhibitor (21). Finally, the monoclonal antibodies used in this project were also characterized for performance in traditional immunoassay applications and made available to the research community through the National Cancer Institute’s Clinical Proteomic Tumor Analysis Consortium (CPTAC) Assay Portal (22, 23) (assays.cancer.gov) and Antibody Portal (antibodies.cancer.gov).

## 2 Methods

### 2.1 Materials and reagents

Urea (#U0631), Trizma base (#T2694), citric acid (#C0706), dimethyl sulfoxide (DMSO, #D2438), EDTA (#E7889), EGTA (#E0396), and iodoacetamide (IAM, #A3221) were obtained from Sigma (St. Louis, MO, USA). Acetonitrile (MeCN, #A955), water (#W6, LCMS Optima^®^ grade), trifluoroacetic acid (TFA, LC-MS grade, #85183), tris(2-carboxyethyl)phosphine (TCEP, #77720), phosphate-buffered saline (PBS, #BP-399-20), ammonium bicarbonate (A643-500), xylene (#422685000), and (3-[(3-cholamidopropyl) dimethylammonio]-1-propanesulfonate) (CHAPS, #28300) detergent were obtained from Thermo Fisher Scientific (Waltham, MA, USA). RapiGest (#186001861) was obtained from Waters (Milford, MA, USA). Formic acid (#1.11670.1000) was obtained from EMD Millipore (Billerica, MA, USA). Lys-C (Wako, Richmond, VA, USA, #129-02541), trypsin (Worthington, Lakewood, NJ, USA #LS003740), and sequencing grade trypsin (Promega, Madison, WI, USA, #V5111) were used for the digestion of samples. Rabbit monoclonal antibodies (mAbs) were acquired from Epitomics/Abcam (Cambridge, MA, USA) and Excel Biopharm (Burlingame, CA, USA). Mouse monoclonal antibodies were acquired from Precision Antibody (Columbia, MD, USA) and the Antibody Development Facility at the Fred Hutchinson Cancer Center (Seattle, WA, USA). Light (unlabeled) synthetic peptides were obtained from Vivitide (Gardner, MA, USA) as crude (flash purified) grade. Cleavable stable isotope-labeled (heavy) peptides containing additional amino acids on the ends of tryptic cut sites were obtained from Vivitide and were purified >95% by HPLC, labeled with [^13^C and ^15^N] at the tryptic C-terminal Arg or Lys, and quantified by amino acid analysis (AAA). Aliquots of the peptide standards were stored in 5% acetonitrile/0.1% formic acid at −80°C until use.

### 2.2 Cell lines, culture conditions, and cell lysis

HeLa [American Type Culture Collection (ATCC, Manassas, VA, USA), #CCL-2], Jurkat (ATCC, #TIB-152), A549 (ATCC, #CCL-185), MCF7 (ATCC, #HTB-22), and NCI-H226 (ATCC, # CRL-5826) cell lines were cultured and harvested as previously described (12).

### 2.3 Human samples

Plasma and serum were collected from two phase II clinical trials of pembrolizumab, the first for previously untreated follicular lymphoma and the second for relapsed/refractory mycosis fungoides and Sezary syndrome, as previously described (21). Secondly, tissue samples used for the evaluation of the assay in formalin-fixed paraffin-embedded (FFPE) samples were collected from patients with untreated diffuse large B-cell lymphoma prior to receiving pembrolizumab with chemoimmunotherapy. The studies were approved by the Fred Hutchinson Cancer Center—University of Washington Consortium Institutional Review Board, all patients provided written informed consent to participate, and the samples for research were fully de-personalized [PIs: Ajay Gopal, M.D. (9975); Stephen D. Smith, M.D. (9291); or Youn Kim, M.D. (CITN-10)]. The plasma matrix used in the validation studies was obtained from BioIVT (Westbury, NY, USA). Frozen tissue and plasma samples for the determination of detectability were supplied by the Clinical Proteomics Tumor Analysis Consortium (CPTAC) as anonymized samples from consenting donors collected under IRB-approved protocols (12). Tissue subcompartment cellularity (e.g., tumor, stroma, adipocytes, lymphocytes) was calculated using the HALO Tissue Classifier (Indica Labs, NM, USA) as previously described (18).

### 2.4 Sample analysis by immuno-MRM

Protein extraction, digestion, immuno-affinity enrichment, and analysis by liquid chromatography–multiple reaction monitoring were conducted as previously described (12, 13) with the following modifications. Frozen tissue lysates were cleared by centrifugation at 20,000×*g* for 10 min at 4°C, and protein concentration was determined using a Micro BCA Protein Assay Kit (Pierce, Rockford, IL, USA, #23235) prior to storage in liquid nitrogen until digestion. Digestion of plasma was performed with Lys-C for 2 h at 37°C [1:50 (w/w) enzyme:substrate ratio] followed by trypsin (Worthington, #LS003740) for overnight digestion at 37°C [1:50 (w/w) enzyme:substrate ratio]. Enrichments were performed using a mixture of 45 antibodies (four capture more than one peptide) coupled to protein G magnetic beads (GE Sepharose, #28-9513-79) at a 5-μg antibody:1-μl bead slurry ratio. For liquid chromatography, the trap and analytical columns were heated at 45°C and interfaced to the mass spectrometer using a CaptiveSpray nano electrospray source (Bruker, Billerica, MA, USA).

### 2.5 Data analysis

MRM data were analyzed as previously described (12) using Skyline (24, 25), a software platform for targeted proteomics and data are made publicly available through Panorama Public (https://panoramaweb.org/IO2immunoMRMpanel.url) (26).

Minimum tissue requirements were predicted by comparing the signal-to-noise measurements made at 500-μg input of frozen tissue to the lower limit of quantification (LLOQ) for each analyte. Comparisons for decreasing input mass levels were evaluated using a one-sample *t*-test, and those signals with 95% confidence above the LLOQ were considered detectable. For the minimum tissue calculated, the confidence interval was based on the standard deviation of the signal-to-noise distribution and degrees of freedom (equal to the number of samples for each tissue site − 1).

For the analysis of correlative biomarkers, we combined the results from IO-1 and IO-2 panels. We applied log_2_ transformation of the peak area ratio values and filtered out 39 peptides that had missing values (i.e., below LLOQ) in more than 105 samples. Additionally, four samples that had missing values in more than 70 peptides were removed. The remaining missing values in the data were imputed with LLOQ/3. Peak area ratios for each peptide were normalized to have mean = 0 and standard deviation = 1 for input to the following linear mixed-effect regression model:

Log peak ratio ~ time + plasma/serum + response category (CR/PR or SD/PD) + 1/Subject,

where CR = complete response, PR = partial response, SD = stable disease, and PD = progressive disease. Three time points were considered: pretreatment (Pre), cycle 2 (C02), and end of treatment (EOT). The absolute time range of EOT in relation to C02 varied for each individual patient (i.e., individuals who came off treatment at different time points in the study).

### 2.6 Fit-for-purpose assay validation

Assay validation was conducted as previously described (12) with the following modifications. For the response curves, cleavable heavy stable isotope-labeled peptides were added to aliquots of the background matrix (150 μg of tissue, 10 μl of plasma) at the following concentrations: 13,333, 1,333, 133, 53, 21, 8.5, 3.4, and 1.4 fmol/mg for tissue lysates and 200, 20, 2, 0.8, 0.32, 0.128, 0.0512, and 0.02048 fmol/μl for plasma. Light peptide was also added at 133 fmol/mg for tissue lysates and 20 fmol/μl for plasma. Repeatability standards were evaluated at three levels (low, medium, and high) using 133, 1,333, and 13,333 fmol/mg of cleavable peptide and 1,333.3 fmol/mg of light peptide for the tissue samples and 2, 20, and 200 fmol/μl of cleavable peptide and 20 fmol/μl of light peptide for the plasma samples. The reproducibility of measuring endogenous proteins was characterized by using the same matrix used to generate the response curves and repeatability studies. Cleavable heavy peptide standards were spiked at 1,333 fmol/mg for tissue and 20 fmol/μl for plasma samples. Five aliquots were measured in complete process replicates (including digestion, capture, and mass spectrometry) over five independent days (total of 25 replicates). All data points were required to be >LLOQ. To measure the stability of the enriched peptides, heavy peptide standards (1,333 fmol/mg for tissue and 20 fmol/μl for plasma) were added to aliquots of the same matrix used in the response curves and analyzed after storage at 4°C in the autosampler for approximately 24 h and after two freeze–thaw cycles. Each condition was measured in process triplicate. Data points <LLOQ were filtered from the analysis. Finally, dilution linearity was characterized by measuring endogenous protein in serial dilution of a pool of cell line lysates from the following cell lines (relative contribution in parentheses): MCF10A (5), H226 (3.75), H2122 (3), H2444 (3), COR-L23 (2.5), COLO205 (2), HEPG2 (2), H1792 (1.25), K-562 (1.25), T47D (1.25), and CCRF-CEM (1). The pooled lysate was diluted two-fold in lysis buffer to obtain the following range of total protein inputs: 500, 250, 125, 62.5, 31.25, 15.63, 7.81, and 3.9 μg. Heavy cleavable standards were spiked into diluted lysates at 200 fmol per aliquot. Samples were processed in triplicate according to the trypsin digestion and immunoaffinity workflows described above.

### 2.7 Characterization of antibodies

The performance of the antibodies generated in this study was characterized in traditional Western immunoblotting, automated Western blot, reverse-phase protein array, and immunohistochemistry as previously described (12), with the following modifications. In Western blotting, whole cell lysates were diluted to 1 mg/ml in reducing conditions (20 ml, 20 μg of total protein/lane), and secondary HRP-linked rabbit-specific antibody (Jackson ImmunoResearch, West Grove, PA, USA, 111-035-144) or secondary HRP-linked mouse antibody (Jackson ImmunoResearch Laboratories, 115-035-062) was used. For immunohistochemistry, tissue microarrays (TMA, MTA-6A) consisting of breast, ovary, colon, and lung tumors were constructed using a tissue arrayer (Pathology Devices, Westminster, MD, USA). Tissue cores of 1.0 mm diameter were arrayed on a recipient paraffin block, with a representative tumor area carefully selected from a hematoxylin and eosin (H&E)-stained section of a donor block for each tumor. Immunohistochemical staining was performed according to the protocol detailed in the CPTAC Antibody Portal (antibodies.cancer.gov) SOP M-106. Briefly, TMA blocks were cut into 5-µm-thick sections, deparaffinized through xylene, and rehydrated with graded alcohols to distilled water. Antigen retrieval was performed in a pressure cooker (Pascal; Dako, Carpinteria, CA, USA) with pH 6.0 citrate buffer (Dako) for 20 min. Endogenous peroxidase activity was blocked with 3% H_2_O_2_ for 10 min and incubated with an additional protein block (Dako) to abate non-specific staining. Subsequently, primary antibody hybridization was carried out at an optimized dilution for 60 min at room temperature. Sections were labeled with an EnVision+ detection system (Dako) for 30 min and then visualized using 3,3′-diaminobenzidine (Dako, #K3468). All sections were counterstained with hematoxylin (StatLab Medical, Columbia, MD, USA) and coverslipped after dehydration.

## 3 Results

### 3.1 Method development

We developed a multiplexed quantitative assay (the “IO-2 assay”) for measuring immunomodulatory proteins in clinical biospecimens. The target immune-related proteins were selected by searching the literature for immunomodulatory proteins (**Supplementary Table 1**) and by consultation with academic and industrial experts in mmune-oncology. Proteotypic surrogate peptides for the target proteins were selected using empirical evidence from proteomic datasets (27–39) and established selection procedures (40). Briefly, candidate peptides were ranked according to detection frequency and/or measured intensity in proteomic datasets, physical properties (e.g., length, position in protein), and chemical properties (e.g., amino acid composition, hydrophobicity). Peptides with frequent variant sites or posttranslational modifications (PTMs) were not selected, except for specific phosphorylation events that were targeted. A panel of 49 peptides (43 proteins), including three phosphorylation sites, was selected for assay development (**Table 1**).

**Table 1:** Proteins and peptides targeted in the “IO-2” immuno-MRM assay panel.

Gene symbol	Peptide modified sequence [Cam] = carbamidomethylation [Ox] = oxidation [PO4] = phosphorylation	Label	CPTAC Antibody Portal ID (antibodies.cancer.gov)	CPTAC Assay Portal ID (assays.cancer.gov)
ALCAM	ALFLETEQLK	ALCAM_ALFL	CPTC-ALCAM-2	CPTAC-6040
ARG1	DVDPGEHYILK	ARG1_DVDP	CPTC-ARG1-1	CPTAC-6041
BAX	MIAAVDTDSPR	BAX_MIAA	CPTC-BAX-1	CPTAC-6042
BAX	M[Ox]IAAVDTDSPR	BAX_MIAAox	CPTC-BAX-1	CPTAC-6035
BCL10	GLDTLVESIR	BCL10_GLDT	CPTC-BCL10-1	CPTAC-6057
BTK	ELGTGQFGVVK	BTK_ELGT	CPTC-BTK-1	CPTAC-6062
BTLA	EAPTEYASIC[Cam]VR	BTLA_EAPT	CPTC-BTLA-3	CPTAC-6043
CD38	VQTLEAWVIHGGR	CD38_VQTL	CPTC-CD38-1	CPTAC-6063
CD4	SLWDQGNFPLIIK	CD4_SLWD	CPTC-CD4-1	CPTAC-6039
CD48	LQVLDPVPKPVIK	CD48_LQVL	CPTC-CD48-1	CPTAC-6064
CSF2RA	NTQPGTENLLINVSGDLENR	CSF2RA_NTQP	CPTC-CSF2RA-1	CPTAC-6044
ERCC2	EVPLPAGIYNLDDLK	ERCC2_EVPL	CPTC-ERCC2-1	CPTAC-6071
FOXO1	ASLQSGQEGAGDSPGSQFSK	FOXO1_ASLQ	CPTC-FOXO1-1	CPTAC-6045
GAPDH	GALQNIIPASTGAAK	GAPDH_GALQ	CPTC-GAPDH-1	CPTAC-3842
HLA-DRA	EPNVLIC[Cam]FIDK	HLA-DRA_EPNV	CPTC-HLA-DRA-1	CPTAC-6047
HLA-E	SWTAVDTAAQISEQK	HLA-E_SWTA	CPTC-HLA-E-1	CPTAC-6048
IDO1	HLPDLIESGQLR	IDO1_HLPD	CPTC-IDO1-3	CPTAC-6072
IFIT1	VLDQIEFLDTK	IFIT1_VLDQ	CPTC-IFIT1-1	CPTAC-6065
IFIT2	AIHHFIEGVK	IFIT2_AIHH	CPTC-IFIT2-1	CPTAC-6055
IFIT3	QAEELIQQEHADQAEIR	IFIT3_QAEE	CPTC-IFIT3-1	CPTAC-6049
IL16	LLSTQAEESQGPVLK	IL16_LLST	CPTC-IL16-1	CPTAC-6050
IL1A	ANDQYLTAAALHNLDEAVK	IL1A_ANDQ	CPTC-IL1A-1	CPTAC-6051
IL2RG	GLAESLQPDYSER	IL2RG_GLAE	CPTC-IL2RG-1	CPTAC-6066
IL6R	SPLSNVVC[Cam]EWGPR	IL6R_SPLS	CPTC-IL6R-1	CPTAC-6036
ITGAX	GVQSLVLGAPR	ITGAX_GVQS	CPTC-ITGAX-1	CPTAC-6067
LGALS9	NLPTINR	LGALS9_NLPT	CPTC-LGALS9-1	CPTAC-6052
LY75	AANDPFTIVHGNTGK	LY75_AAND	CPTC-LY75-1	CPTAC-6059
MCL1	VARPPPIGAEVPDVTATPAR	MCL1_VARP	N/A	CPTAC-6073
MGMT	GNPVPILIPC[Cam]HR	MGMT_GNPV	CPTC-MGMT-1	CPTAC-6068
MKI67	DINTFLGTPVQK	MKI67_DINT	CPTC-MKI67-3	CPTAC-5908
MKI67	DINTFLGT[PO4]PVQK	MKI67_pT_DINT	CPTC-MKI67-3	CPTAC-5909
MKI67	NINTFVET[PO4]PVQK	MKI67_pT_NINT	CPTC-MKI67-4	CPTAC-5910
MPO	IGLDLPALNMQR	MPO_IGLD	CPTC-MPO-2	CPTAC-6053
MPO	IGLDLPALNM[Ox]QR	MPO_IGLDox	CPTC-MPO-2	CPTAC-6037
MSH6	LANLPEEVIQK	MSH6_LANL	CPTC-MSH6-1	CPTAC-6060
MST1	VVGGHPGNSPWTVSLR	MST1_VVGG	CPTC-MST1-1	CPTAC-6061
PMS1	LDELLQSQIEK	PMS1_LDEL	CPTC-PMS1-1	CPTAC-6074
POLD1	EVSHLNALEER	POLD1_EVSH	CPTC-POLD1-1	CPTAC-6058
PVR	HGESGSMAVFHQTQGPSYSESK	PVR_HGES	CPTC-PVR-1	CPTAC-6038
PVR	HGESGSM[Ox]AVFHQTQGPSYSESK	PVR_HGESox	CPTC-PVR-1	CPTAC-6034
RB1	IPGGNIYISPLK	RB1_IPGG	CPTC-RB1-3	CPTAC-3251
RB1	IPGGNIYIS[PO4]PLK	RB1_pS_IPGG	CPTC-RB1-2	CPTAC-3288
SIGLEC1	LHAEPVPTLAFTHVAR	SIGLEC1_LHAE	CPTC-SIGLEC1-1	N/A
SIGLEC7	FHLLGDPQTK	SIGLEC7_FHLL	CPTC-SIGLEC7-1	CPTAC-6056
SPP1	GDSVVYGLR	SPP1_GDSV	CPTC-SPP1-1	CPTAC-6075
TAP1	ELISWGAPGSADSTR	TAP1_ELIS	CPTC-TAP1-1	CPTAC-6069
TAPBP	IHHPSLPASGR	TAPBP_IHHP	CPTC-TAPBP-2	CPTAC-6076
XRCC5	TLFPLIEAK	XRCC5_TLFP	CPTC-XRCC5-1	CPTAC-6070
ZAP70	TVYHYLISQDK	ZAP70_TVYH	CPTC-ZAP70-1	CPTAC-6054

Two custom reagents were required for assay development: anti-peptide antibodies and synthetic peptides. Custom anti-peptide monoclonal antibodies to the linear peptide sequences in **Table 1** were generated as previously described (41). We generated 45 custom monoclonal anti-peptide antibodies for the IO-2 multiplexed immuno-MRM assay. Unlabeled (i.e., light) tryptic peptides were synthesized for assay development, and cleavable heavy stable isotope-labeled peptides (42) were synthesized to be used as internal standards. The cleavable standards use the native protein sequence to incorporate two to five additional amino acids on either side of the trypsin cleavage sites. Using cleavable standards allows for the addition of standards prior to digestion and provides a “within-sample” control for trypsin digestion (42). The synthetic light peptides were used to select transitions (precursor and fragment ion pairs specific for each peptide of interest), determine the chromatographic retention time of each peptide, and optimize collision energy parameters in the mass spectrometer.

### 3.2 Analytical method validation of the multiplexed IO-2 assay panel

**Figure 1:**
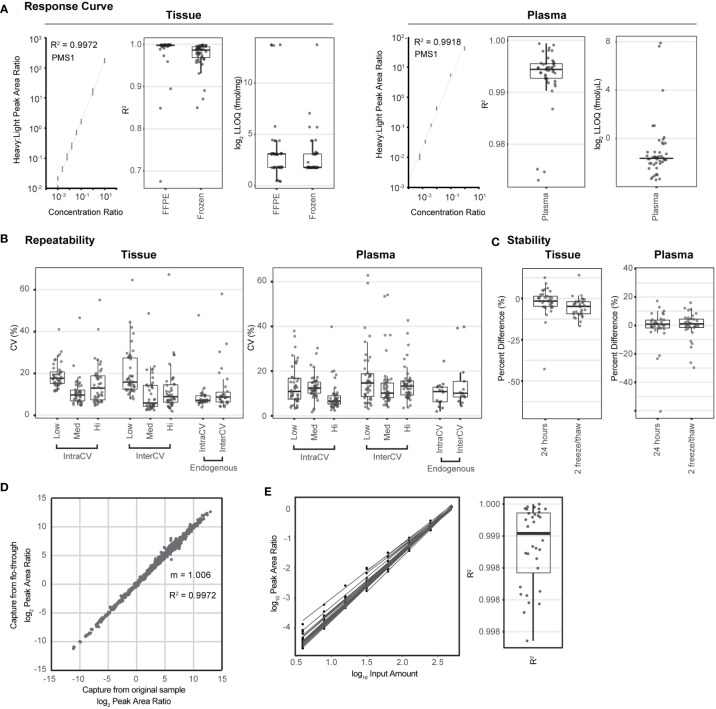
The multiplexed assay panel was characterized according to fit-for-purpose validation guidelines. **(A)** Figures of merit for assays characterized by response curves in tissue and plasma matrices. Representative response curves for the peptide LDELLQSQIEK from PMS1 are shown in tissue lysate and plasma. Distribution of correlation coefficients (*R* ^2^) and lower limits of quantification (LLOQs) determined in FFPE tissue, frozen tissue, and plasma matrices. **(B)** Repeatability characterized in tissue and plasma. For each matrix, within-day (IntraCV) and between-day (InterCV) repeatability of assays are shown measured at three concentrations (Low, Med, Hi) covering the linear range. Each point is the %CV of triplicates measured over 5 days (*n* = 15 at each concentration for a peptide). Endogenous measurements refer to the within-day (IntraCV) and between-day (InterCV) variability of endogenous peptides detected above the LLOQ in five replicates measured over 5 days (*n* = 25). **(C)** Stability shows the distribution of relative percent difference for two conditions: i) stored for 24 h at 4°C and ii) after two freeze–thaw cycles, compared with the immediate analysis. **(D)** Correlation plot for the results from sequential enrichment of analytes (captured from the flow-through) *versus* direct enrichment (captured from the original sample) using frozen lung tissue lysate. **(E)** Distribution of correlation coefficients (*R* ^2^) for peptides detected above the LLOQ in the linear dilution of cell lysate. Dilution curves are plotted using the log(10) Peak Area Ratio (light:heavy) *versus* log(10) Input Amount. For box plots, the line shows the median value, boxes show the interquartile ranges, and the whiskers show 5%-95% of data.

We used published guidelines (20, 22, 43) to perform fit-for-purpose method validation to characterize the performance (i.e., linearity, limits of quantification, repeatability, and stability) of the multiplexed IO-2 immuno-MRM assay panel in tissue and plasma matrices. The response curves were used to determine the linear ranges and limits of quantification (LOQs) using pooled background matrices of protein lysates from three human biospecimen types: i) frozen lung tumor tissues, ii) FFPE lung tumor tissues, and iii) plasma. Peak area ratios (heavy:light) were plotted as a function of analyte concentration to determine the assay figures of merit in the response curves. Representative response curves measured in tissue and plasma are shown in **Figure 1A**. LLOQs were determined by the lowest point with CV <20%. Linear ranges were determined by using points on the linear regression with squared correlation coefficients (*R* ^2^) greater than 0.85. The linear range was a minimum estimate for assays where the highest concentration point was still linear. Figures of merit for each peptide are plotted in **Figure 1A** and reported in **Supplementary Table 2**. The median linear dynamic range was ≥2.8 orders of magnitude in all matrices with median LLOQ 8.5 fmol/mg (range 1.4–13,000 fmol/mg) in tissues and 0.32 fmol/µl (range 0.13-200 fmol/µl) in plasma. The characterized LLOQs were slightly lower in the frozen tissue compared with the FFPE tissue matrix.

Intra-assay (within-day) and inter-assay (between-day) repeatability were characterized by performing complete process measurements over 5 days. First, repeatability was characterized over the linear range by spiking heavy cleavable peptide standards into 150 µg of aliquots of the pooled tissue lysate or 10 µl of aliquots of the plasma matrix at three amounts (20, 200, 2,000 fmol; low, medium, high) with the addition of equal amounts of light peptides (200 fmol). The spiked samples were processed in triplicate over 5 days. For tissue, the median intra-assay variability was 17.2%, 9.5%, and 10.3% for the low- to high-concentration samples, and the median inter-assay variability was 15.6%, 7.0%, and 9.2% (low to high) (**Figure 1B**, **Supplementary Table 2**). One peptide, SIGLEC1.LHAEPVPTLAFTHVAR, failed validation, likely due to poor enrichment of the target peptide leading to low intensity of all signals, even at the highest concentration attempted. Two assays were outliers in more than one condition (oxidized methionine MPO.IGLDLPALNM(ox)QR and SPP1.GDSVVYGLR), showing high intra- and inter-assay variability in the tissue samples. Variability for MPO.IGLDLPALNM(ox)QR is likely due to the inconsistency of the oxidized methionine. Repeatability for the unmodified version of the peptide IGLDLPALNMQR was all below 18% CV. For measurements made in the plasma matrix, the median intra-assay variability was 13.1%, 14.5%, and 9.0% for the low- to high-concentration samples and the median inter-assay variability was 16.8%, 12.4%, and 15.4% for the low- to high-concentration samples (**Figure 1B**, **Supplementary Table 2**). Three assays (oxidized methionine BAX.M(ox)IAAVDTDSPR, MGMT.GNPVPILIPCHR, and ERCC2.EVPLPAGIYNLDDLK) showed high variability when applied to plasma. BAX.M(ox)IAAVDTDSPR also showed high instability in plasma (see below), indicating that high variability is likely due to the oxidation of methionine. Similar to the peptide for MPO above, the unmodified form of the peptide was measured with expected precision. High imprecision for MGMT.GNPVPILIPCHR was likely due to a low signal (close to the LLOQ) in plasma. Phosphorylated targets to MKI67 and RB1 failed validation in plasma due to no signal detected.

In addition to the repeatability of spiked-in peptides, the intra-assay and inter-assay repeatability for the detection of endogenous protein were characterized. Cleavable heavy peptide standards were added into 150 µg of aliquots of a pooled frozen tissue lysate and 10 µl of aliquots of plasma, and measurements were made using five complete process replicates over 5 days (*n* = 25). Thirty peptides were detected above the LLOQ in the endogenous tissue sample. The median intra-assay variability for endogenous detection was 7.1% (range 3.8%-48%), and the median inter-assay variability was 8.7% (range 4.0%-58%) (**Figure 1B**, **Supplementary Table 2**). Again, the MPO.IGLDLPALNM(ox)QR peptide was a clear outlier and showed high irreproducibility in tissue. Twenty peptides were detected above the LLOQ in the endogenous plasma sample. The median intra-assay variability was 10.8% (range 1.9%-24%) and the median inter-assay variability was 10.1% (range 4.4%-40%), shown in **Figure 1B**. Peptides MPO.IGLDLPALNMQR and BAX.MIAAVDTDSPR were outliers in interday plasma endogenous variability, and both contain a methionine residue, indicating that there may be interday imprecision due to oxidized methionine. However, the oxidized forms of the peptides were not detected.

The stability of the processed samples was evaluated by analyzing 150 µg of aliquots of the same tissue matrix used in the curves or 10 µl of aliquots of the same plasma matrix. Cleavable standards were added at 200 fmol per aliquot. The processed samples were analyzed under two conditions: i) 4°C on the autosampler for 24 h and ii) after two freeze–thaw cycles; the control samples were analyzed immediately after capture. Each test case was measured and processed in triplicate. The percent differences, comparing the peak area ratios (light:heavy) between the control samples and samples with different handling conditions, were used to evaluate peptide stability. The median percent difference relative to the fresh sample ranged from −1.6% to −4.8% in tissue and 0.9% to 1.1% in plasma, indicating acceptable stability for most peptides (**Figure 1C**, **Supplementary Table 2**). In tissue, the peptide GAPDH.GALQNIIPASTGAAK showed instability at the 24-h time point, indicating that it should be analyzed immediately. In plasma, there were three peptides that were consistent outliers, namely, BAX.M(ox)IAAVDTDSPR, MPO.IGLDLPALNM(ox)QR, and LGALS9.NLPTINR, indicating that these peptides should be analyzed immediately following capture.

We previously demonstrated the sequential application of immuno-MRM assays to the same biospecimen, sparing precious samples. Specifically, the flow-through from the antibody capture step of the first assay is subjected to immunoaffinity enrichment of different targets with another panel of antibodies (15, 17). This ability to perform sequential immuno-captures enables the analysis of more targets in a single biospecimen. We validated the performance of the IO-2 panel in the flow-through of samples first analyzed using the IO-1 assay. Using aliquots of a frozen tissue lysate spiked with cleavable heavy standards for the IO-1 and IO-2 panels, we compared the results from direct enrichment of the IO-2 assay to those from sequential capture using the flow-through from the IO-1 assay panel; the experiment was performed in triplicate. **Figure 1D** shows the correlation of direct measurements to those made using the flow-through (sequential). Overall, there was excellent correlation (*R* ^2 = ^0.9972) and agreement (slope = 1.006), confirming that the IO-2 panel can be used sequentially with the IO-1 panel on a single biospecimen, expanding the number of analytes that can be measured in a single biospecimen.

Finally, because clinical biospecimens may have limited and variable material for analysis, we characterized the ability to vary protein input amounts and normalize results by the input mass through a dilutional linearity experiment. A pooled background of frozen tissue lysate was diluted in a digestion buffer over a range of 3.9-500 µg of input protein lysate, and each dilution point was processed in triplicate. Peak area ratios (light:heavy) for 32 endogenous peptides detected above the LLOQ were plotted as a function of input lysate (**Figure 1E**). Regression coefficients show high linearity over the range of inputs (median *R* ^2 = ^0.9991; **Figure 1E**), indicating that peak area ratios can be normalized to protein input.

### 3.3 Determination of assay utility in biospecimens

We next applied the multiplex assay to a panel of tumor tissue biopsy specimens with two aims: i) to evaluate the utility of the assay for measuring endogenous levels of analytes in clinical biospecimens and ii) to determine sample requirements for analyte detection in tissue where clinical material may be limited.

**Figure 2:**
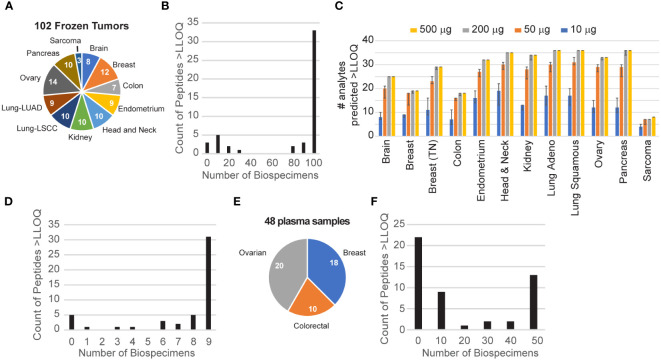
Utility of endogenous protein quantification in tissues and plasma. **(A)** Frozen tissues were obtained for 102 tumors from 11 tumor types. The number of each type is indicated in the pie chart. **(B)** Distribution of peptide detection plotted as a histogram, showing the number of peptides detected above the LLOQ across the 110 frozen tumors. **(C)** The number of analytes predicted to be detectable for decreasing amounts of tissue was extrapolated from the signal-to-noise ratio measured using 500 μg of protein digest input. Error bars show the 95% confidence interval. **(D)** Distribution of peptide detection plotted as a histogram, showing the number of peptides detected above the LLOQ across the nine FFPE tumors. **(E)** Plasma samples were obtained for 48 patients with breast, colorectal, or ovarian tumors, as indicated in the pie chart. **(F)** Distribution of peptide detection plotted as a histogram, showing the number of peptides detected above the LLOQ across the 45 plasmas using 100 μl of aliquots of plasma as input.

The tissue panel included 102 frozen biopsy specimens collected from 11 different tumor types, namely, brain, breast, colorectal, endometrium, head and neck, kidney, lung (adenocarcinoma and squamous cell carcinoma), ovarian, pancreas, and soft tissue sarcoma (**Figure 2A**) with a median percentage of tumor cells >50% (12). Each biospecimen (500 µg of aliquots of the frozen tissue lysates) was independently analyzed using the IO-2 assay. The specificity of endogenous peptide detection was assured by equal retention times and relative areas of light and heavy transitions. Overall, 46/49 peptides corresponding to 41 proteins were detected above the LLOQ in the frozen tissue biospecimens. Three peptides were not detected in the biospecimen panel: SIGLEC1.LHAEPVPTLAFTHVAR, SIGLEC7.FHLLGDPQTK, and phospho-threonine MKI67.NINTFVET(PO4)PVQK. This is not surprising because SIGLEC1 failed validation and SIGLEC7 and phospho-MKI67 were also not detected endogenously in assay validation (**Supplementary Table 2**). A histogram of peptide detection shows that 33 peptides were detected in all samples, with an additional 11 peptides detected above the LLOQ in less than a fifth of the samples (**Figure 2B**).

To guide sample requirements for the detection of endogenous protein in future studies, we used the results of the frozen tissue array to estimate the minimum amount of tissue needed to detect each analyte at endogenous levels. **Figure 2C** shows the number of peptides predicted to be detected for tissue inputs ranging from 10 to 500 µg. For each tumor site, the number of peptides predicted to be detected remains within ~80% of the total, even with 10-fold less input. Consistent with this, despite sample inputs ranging from 2- to >10-fold less material compared with frozen tissue, we successfully quantified 41/49 peptides above the LLOQ in protein lysates from at least 5 of 9 FFPE lymph node biopsies from patients with untreated diffuse large B-cell lymphoma (**Figure 2D**), demonstrating the utility of the assay in lymphoid tissue and in FFPE biospecimens. This indicates that most of the assays are amenable to a range of biospecimen sizes and input amounts.

Although the immune-related proteins targeted in the IO-2 assay were selected from the tumor microenvironment, a subset is also detectable in the circulation. To evaluate the ability of the assay to measure endogenous protein levels in human plasma, we applied the assays to a panel of 48 plasma samples obtained from patients with three different tumor types (breast, colorectal, ovarian) (**Figure 2E**). Each 100 µl of aliquot of the plasma sample was independently analyzed using the IO-2 assay. The specificity of endogenous peptide detection was assured by equal retention times and relative areas of light and heavy transitions. Overall, 27/49 peptides corresponding to 25 proteins were detected above the LLOQ in the plasma samples (**Figure 2F**).

### 3.4 Proof-of-principle demonstration of correlative studies in clinical trial specimens

To provide a proof-of-principle demonstration for the use of the mass spectrometry-based, immuno-MRM assay panel for correlative studies, we applied the multiplexed assays to serum and plasma samples from two clinical trials. In each demonstration, we coupled the IO-2 assay sequentially with the previously published IO-1 assay (12) on each clinical biospecimen in order to evaluate the advantages of using the assay panels in combination.

**Figure 3:**
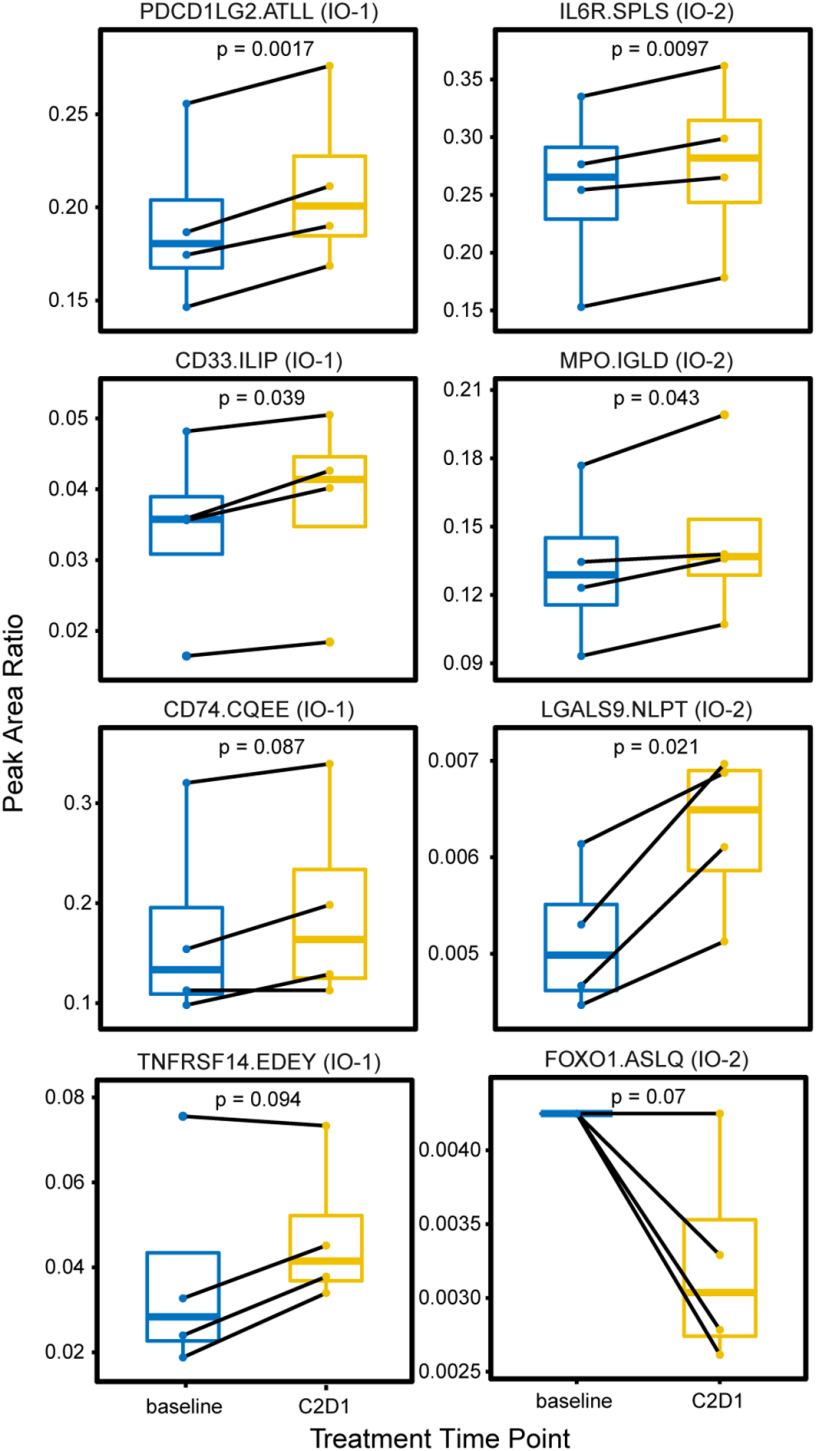
Changes in plasma expression levels following treatment with the immune checkpoint inhibitor pembrolizumab. Plasma was collected from four patients with follicular lymphoma undergoing treatment with an immune checkpoint inhibitor (pembrolizumab) at two time points: prior to treatment (baseline) and on the first day of cycle 2 or treatment (C2D1). Box plots show the distribution of expression, measured as the peak area ratio of light endogenous:heavy internal standard. Pairwise samples for each patient are connected with a solid black line. Peptide labels show the gene symbol followed by the first four amino acids of the peptide sequence; the assay panel is indicated in parentheses. Solid horizontal lines indicate the median peak area ratio, boxes show the interquartile range, and vertical lines show the 5-95^th^ percentiles. Significance is indicated by *p*-values from a two-sided pairwise *t*-test.

In the first study, longitudinal plasma samples were collected from four patients enrolled in a phase 2 trial of an immune checkpoint inhibitor (pembrolizumab) for previously untreated follicular lymphoma. The samples were collected at baseline and following cycle 2 of pembrolizumab. One hundred microliters of aliquots of plasma were sequentially processed using the IO-1 and IO-2 multiplexed assays. Overall, 65/101 peptides corresponding to 61 proteins were detected >LLOQ in at least one sample. A pairwise comparison of the expression levels measured in cycle 2 samples compared with baseline showed eight proteins with significant differences over time (**Figure 3**). An equal number of proteins originating from the IO-1 (PDCD1LG2, CD33, CD74, TNFRSF14) and IO-2 panels (IL6R, MPO, LGALS9, FOXO1) were significantly different, demonstrating the value of combining the panels. Because this experiment was conducted in an early-phase clinical trial with a limited set (*n* = 4) of patients, further studies are necessary to determine the clinical significance of these differences. These results demonstrate the ability of the multiplexed assays to quantify proteins showing differential expression in support of exploratory correlative studies.

We next evaluated the use of the IO assay panels for correlative studies in a larger trial. We analyzed samples from the Cancer Immunotherapy Trials Network (CITN-10) phase 2 clinical trial of pembrolizumab for the treatment of refractory or relapsed mycosis fungoides (MF) and Sezary syndrome (SS) (21). MF and SS are common subtypes of cutaneous T-cell lymphomas with poor response rates to systemic therapies. Treatments targeting immune checkpoint molecules, like PD-1, have been associated with 15%-38% overall response rates in MF-type T-cell lymphomas (21, 44). Thus, correlative predictive markers of response to identify patients most likely to respond to targeted therapy would be highly beneficial.

**Figure 4:**
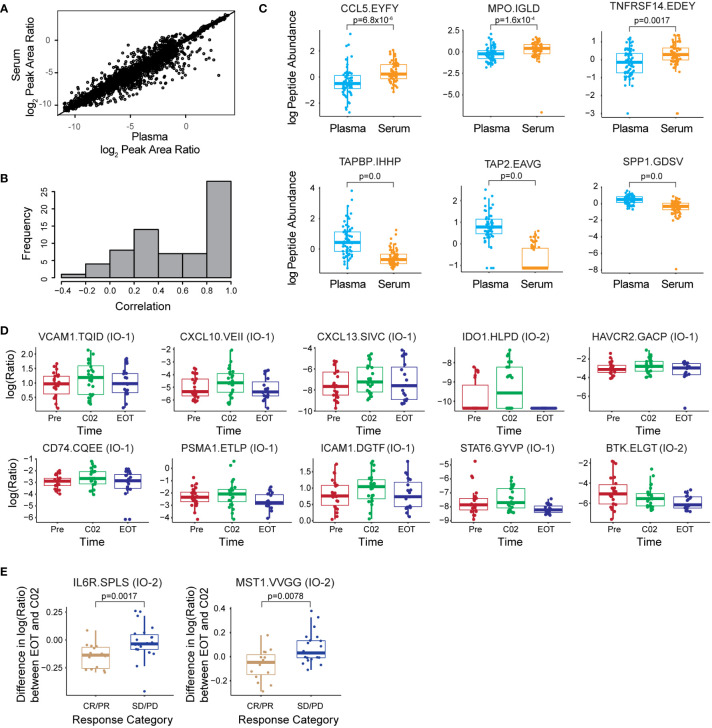
The multiplex assay enables correlative studies in immunotherapy clinical trials. **(A)** Correlation of peak area ratios (light endogenous:heavy internal standard) for 76 peptides over LOQ measured in 67 serum and 67 plasma samples. **(B)** Histogram of the correlation coefficient for each peptide measured in serum and plasma. **(C)** Box plots showing log(2) of peptide abundance for those peptides with differences in serum and plasma. The top 3 peptides elevated in plasma and serum are shown in the plot. **(D)** Box plots showing differences in log(2) peak area ratios for peptides measured longitudinally in plasma. Time points are indicated as prior to treatment (Pre), at cycle 2 of treatment (C02), and end of treatment (EOT). **(E)** Differences in the expression for IL6R and MST1 peptides measured between EOT and administration of C02 depending on the response group [complete/partial remission (CR/PR) and stable/progressive disease (SD/PD)]. Peptide labels show the gene symbol followed by the first four amino acids of the peptide sequence; the assay panel is indicated in parentheses. All box plots show median (horizontal bar), interquartile range (box), and 5-95th percentile (whiskers). Significance is indicated by FDR-adjusted *p*-values.

A total of 134 samples collected from 24 patients were analyzed, consisting of matched serum (*n* = 67) and plasma (*n* = 67) from three time points (pretreatment, cycle 2, and end of treatment), enabling a comparison of assay results between the two biospecimen types. Each sample was sequentially processed using the IO-1 and IO-2 multiplexed assays, using separate 100 µl of aliquots of plasma and serum. Overall, 76/100 peptides corresponding to 72 proteins were detected above the LLOQ in plasma and serum. Three proteins, ANXA1, CD40, and PTPRC, were measured with two peptides, which demonstrated a high correlation (*R* ^2 = ^0.66–0.96) of the peak area ratios (**Supplementary Figure 1**), adding confidence to the protein measurements. In general, the correlation was high between peptide abundances measured in plasma and serum (**Figure 4A**), indicating that either of the sample types may be suitable for correlative studies for most peptides. However, examination of abundances for individual peptides reveals a subset whose abundances in plasma and serum were poorly correlated (**Figure 4B**). The mean peak area ratio for 16/100 peptides differed significantly (adjusted *p*-value < 0.1, **Supplementary Table 3**) between the plasma and serum samples, of which 12 peptides had a higher peak area ratio in the plasma samples, while 4 peptides had a higher peak area ratio in the serum samples. The peptides with the highest differences between plasma and serum were CCL5.EYFYTSGK, MPO.IGLDLPALNMQR, TNFRSF14.EDEYPVGSECCPK, TAPBP.IHHPSLPASGR, TAP2.EAVGGLQTVR, and SPP1.GDSVVYGLR (**Figure 4C**). Differences in protein concentrations between plasma and serum have been described (45).

We next examined whether the assay was able to detect longitudinal changes in protein expression during treatment. Using a linear mixed effects regression model, we identified 10 peptides with significant abundance changes (adjusted *p*-value < 0.05) in plasma and serum across the Pre, C02, and EOT time points (**Supplementary Table 3**, **Supplementary Figure 2**). **Figure 4D** shows the expression level changes across time of the 10 most significant peptides. In general, the expression levels are seen to increase at the C02 treatment time point and decrease to pretreatment levels or lower at the EOT time point. Proteins showing longitudinal changes were split between the IO-1 (VCAM1, CXCL10, CD74, CXCL13, HAVCR2, ICAM1, PSMA1, STAT6) and IO-2 panels (IDO1, BTK), demonstrating the utility of combining the panels.

Next, we used the linear regression model to screen the plasma and serum data for peptides showing different trajectories over the time course of treatment between patients with distinct treatment responses: complete/partial response *versus* stable/progressive disease. Two peptides from the IO-2 panel, corresponding to IL6R and MST1, respectively, showed significant differences between the response classes (**Figure 4E**). The clinical utility of these proteins as predictive biomarkers needs to be validated in larger, follow-up clinical validation studies, but the data support the feasibility of using these new assay panels for correlative studies in clinical trials.

### 3.5 The antibodies are available as a resource to the biomedical community

The novel antibodies generated for the IO-2 immuno-MRM assay panel (as well as for the IO-1 panel; 12) are available as a resource for the research community *via* the CPTAC Antibody Portal (antibodies.cancer.gov). In addition to immuno-MRM, the antibodies were tested for reactivity to the target proteins in conventional immunoassay platforms (**Supplementary Table 4**), including traditional Western blot, automated WES system immunoblot, reverse-phase protein array (RPPA), and immunohistochemistry (IHC). For this set of antibodies, 54% (21/39) of the antibodies tested were positive against recombinant proteins, and 26% (11/42) were positive against lysates (not all antibodies were tested against all cell line lysates). These results were consistent with expectations based on a previous work (46). Based on normalized levels above the background, 17% (7/42) of the antibodies tested were positive in the reverse-phase array. Antibodies scoring positive in Western blotting were tested for application in IHC, with 58% (15/26) of the antibodies testing positive in IHC. The data are summarized in **Supplementary Table 4**, and example images are provided in **Supplementary Figure 3**.

## 4 Discussion

We present the development and characterization of a novel multiplexed immuno-MRM assay panel (IO-2) of 49 assays for quantifying 43 immuno-modulatory proteins in tissue and plasma biospecimens. The assays have been made available to the research community as part of a larger effort under the National Cancer Institute’s Beau Biden National Cancer Moonshot (APOLLO network; 47). The monoclonal antibodies, characterization data, and SOPs are freely accessible to the research community through NCI’s CPTAC Assay Portal (assays.cancer.gov) and CPTAC Antibody Portal (antibodies.cancer.gov).

The panel is an extension of a previous work aimed at enabling multiplex protein quantification of targets related to immuno-oncology. The panel shows excellent quantitative characteristics in tissue and plasma, with a wide dynamic range and high precision and specificity. Furthermore, the panel can be applied in combination with the IO-1 panel (12) by using the flow-through of IO-1 capture for the enrichment of the IO-2 targets. This capability allows for the quantification of 100 peptides corresponding to 86 proteins from a single biospecimen with high precision and specificity. The increased number of proteins measured using the combination of the two panels showed benefit in correlative studies, where the number of targets from both panels was found to be significantly different.

Applied to clinical biospecimens, the assays may support correlative studies, establish metrics of on-target inflammation and tumor response, or provide mechanistic insights to implicate potential new therapeutic targets. We demonstrated the feasibility of applying the assays to a panel of frozen tissue, FFPE tissue, and plasma. A high percentage of the targets (85%-94% in tissue, 55% in plasma) were consistently quantified in biospecimens. In addition, we performed proof-of-principle correlative studies in plasma and serum from clinical trials. Increases in protein expression measured for several immunoregulatory proteins [e.g., VCAM1, CXCL10, CXCL13, HAVCR2 (TIM-3), PDCD1LG2 (PD-L2), TNFRSF14] indicate increased immune and inflammatory activity following treatment. The application of these panels to a wider set of samples may be informative for finding biomarkers of immune-related adverse events in patients receiving immunotherapies. Notably, while these studies were conducted on a limited set of patients, the data identified potential biomarkers of response to therapy for follow-up study in larger clinical validation studies.

Overall, this study highlights the development of a powerful expansion to the quantification of immunomodulatory proteins in clinical biospecimens using mass spectrometry-based assays and provides a suite of novel monoclonal antibodies to the community for implementing the assays.

## Data availability statement

The datasets presented in this study can be found in online repositories. The names of the repository/repositories and accession number(s) can be found in the article. The name of the repository is Panorama, the accession is IO2immunoMRMpanel. The data can be found here: https://panoramaweb.org/IO2immunoMRMpanel.url.

## Ethics statement

The studies involving human participants were reviewed and approved by Institutional Review Board, Fred Hutch Cancer Center - University of Washington Consortium. The patients/participants provided their written informed consent to participate in this study.

## Author contributions

AP conceived or designed the work. JW, LZ, RS, DH, RL, UV, JK, RI, CL, OM, TL, SCo, TC, RR, JK, JR, CP, CR, SH, SCh, JV, PW, AG and SS acquired, analyzed, or interpreted the data. SG-B, WB, SS, AG, NR, SF, PW, JW and AP supervised the studies. JW and AP drafted or substantively revised the work. All authors contributed to the article and approved the submitted version.
