# Supplementary material for: A multiplexed assay for quantifying immunomodulatory proteins supports correlative studies in immunotherapy clinical trials
Source: Front Oncol. 2023 May 2;13:1168710. doi: 10.3389/fonc.2023.1168710 (PMC10185886; doi:10.3389/fonc.2023.1168710)
Supplement: Supplementary file 1 [file DataSheet_1.pdf]

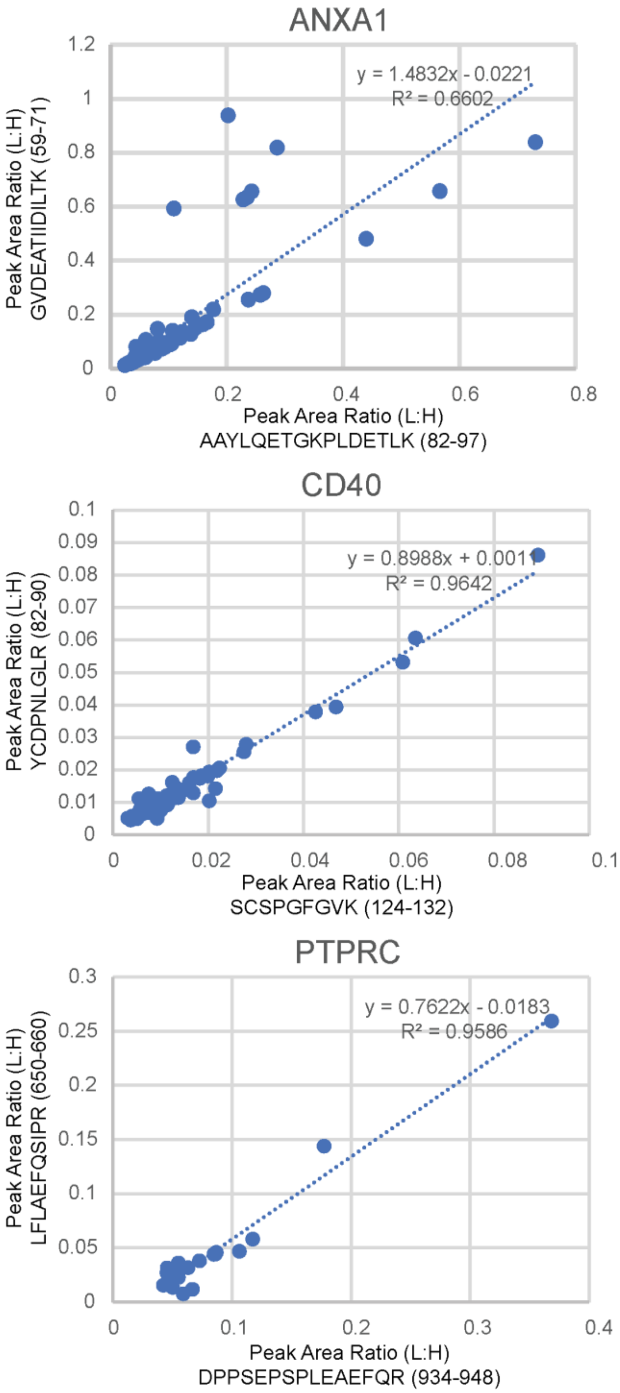

Supplementary Figure 1. Correlation of peak area ratios (Light endogenous : Heavy standard) measured for proteins with two proteotypic peptides in the assay panel. Peptide sequences are labeled on the axes, and the peptide position in the protein is indicated in parentheses. Differences for peptides from ANXA1 were notable in six specimens (110-41-003_C02_Plasma,110-41-004_EOT_Serum,110-18-003_C02_Plasma,110-07-003_C02_Plasma,110-02-001_C02_Plasma). The peptides from ANXA1 do not correspond to differences from known proteoforms and could, for example, represent a post-translational modification.

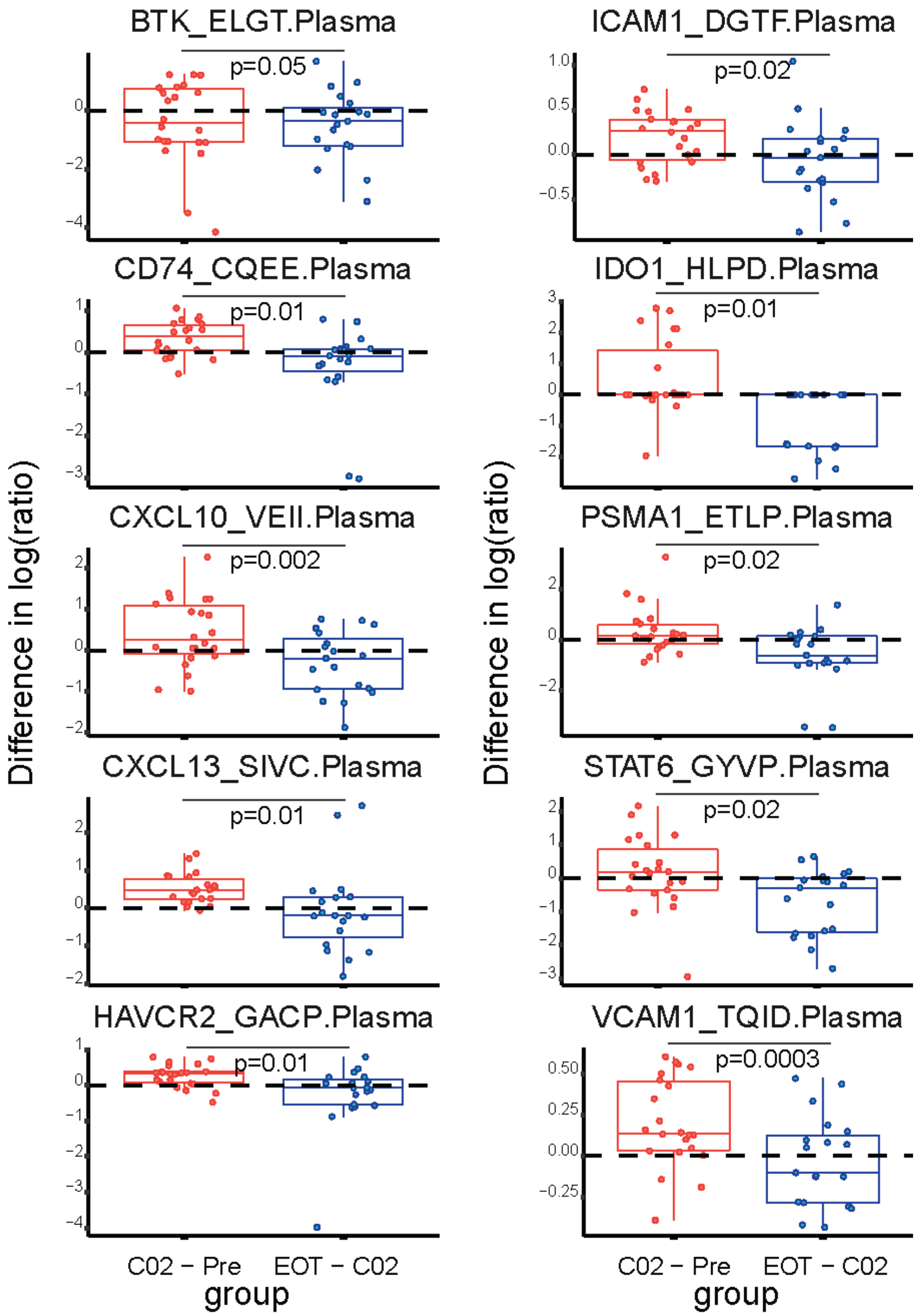

Supplementary Figure 2. Longitudinal changes in protein expression in plasma in patients enrolled in the CITN-10 clinical trial. Time points are indicated as prior to treatment (Pre), at cycle 2 of treatment (C02), and end of treatment (EOT). Box plots show median (horizontal bar), interquartile range (box), and 5-95th percentile (whiskers).

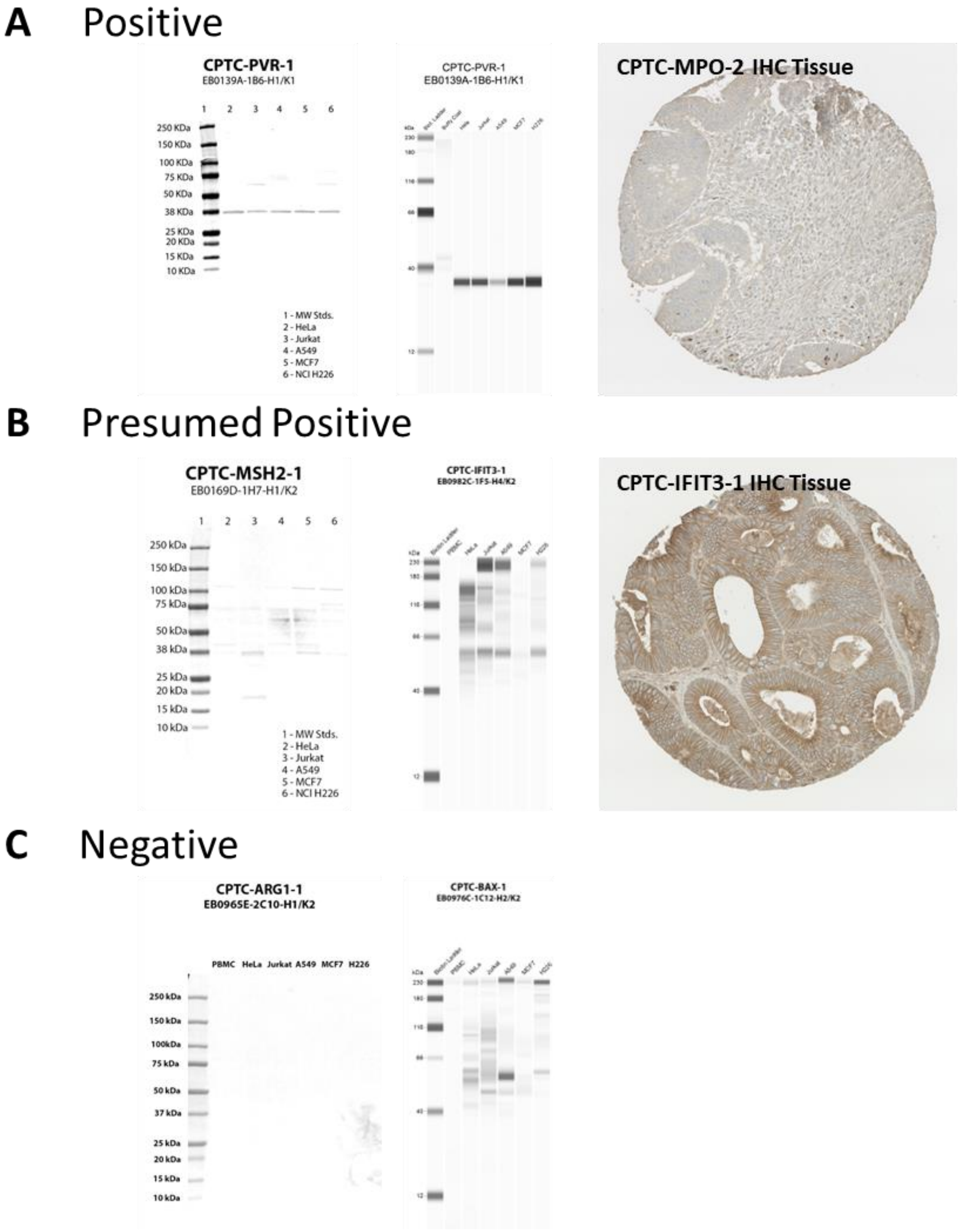

Supplementary Figure 3. Example images for Western Blot, Simple Western, and Immunohistochemistry analysis characterized as Positive (A), Presumed Positive (B), and Negative (C). Positive images show bands at the expected MW in the Westerns and staining in the expected cellular regions. Presumed positive Western images show faint bands at the expected MW along with other bands, and the IHC shows a pattern of staining that is correct but lacks sufficient literature data to confirm cell type specificity. Finally, negative images show the lack of bands at the correct MW. IHC negative results either produce an incorrect staining pattern or an unexpected localization (negative images are read but not digitized/recorded). Images for all analytes are available at antibodies.cancer.gov.
